# Mass Transfer during Atmospheric and Vacuum Frying of Chorizo

**DOI:** 10.1155/2021/9142412

**Published:** 2021-05-22

**Authors:** Piedad Margarita Montero Castillo, Lesly Torres Díaz, Sandy Torres Díaz, Diofanor Acevedo Correa, Raúl J. Martelo Gómez

**Affiliations:** ^1^Grupo de Investigación en Innovación y Desarrollo Agropecuario y Agroindustrial (IDAA), Universidad de Cartagena, Campus Piedra de Bolívar, Av. Consulado, # 48-152, 130015 Cartagena de Indias, Colombia; ^2^Grupo de Investigación en Tecnologías de las Comunicaciones e Informática (GIMATICA), Universidad de Cartagena, Campus Piedra de Bolívar, Av. Consulado, # 48-152, 130015 Cartagena de Indias, Colombia

## Abstract

The main objective of this study was to evaluate the kinetics of moisture and oil uptake during chorizo deep-fat frying as compared to atmospheric and vacuum conditions. The conditions in the process were 90, 120, and 150°C for vacuum frying and 160, 170, and 180°C for atmospheric frying. The kinetics of moisture loss during atmospheric and vacuum frying was studied from the analytical solution of Fick's second law for cylinder geometry. Oil absorption was also determined using a first-order kinetic model. The moisture content decreased by 33.72% at the maximum process temperature and time during vacuum frying (150°C, 360 s), as compared to the atmospheric frying, which was 28.61% (180°C). The oil content at the end of the process (360 s) was 27.79% (90°C), 27.31% (120°C), and 24.82% (150°C) for vacuum-fried chorizos, and higher values were obtained in the atmospheric frying, obtaining values of 34.45% (160°C), 31.36% (170°C), and 28.47% (180°C) (*p* < 0.05). In summary, the vacuum frying yielded sausages with a lower final oil percentage and higher moisture content; these results are promising because they may influence consumer preference for sensory parameters.

## 1. Introduction

Frying is one of the oldest and most popular cooking methods. This process consists of subjecting food to a cooking process by immersion in oil or fat at a temperature above the boiling point of water, normally between 150 and 200°C [1, 2]. Oil absorption is one of the most important quality parameters of fried foods. Oil consumption poses major health problems, such as coronary heart disease, cancer, diabetes, and hypertension [3], and is irreconcilable with consumer awareness of healthier, low-fat food products [4].

Moisture loss and fat absorption are two important mass transports that occur during frying. These two processes can be adjusted by changing the raw material properties and frying conditions to modify the quality and nutritional attributes of fried products [5].

When food is immersed in hot oil, heat transfer occurs by two mechanisms: convection from the oil to the surface of the food and conduction from the surface to the interior of the food [6]. This heat transfer evaporates part of the water present in the food, which escapes to the surface through concentration and pressure gradients. Moisture vaporization influences physical and chemical changes, such as starch gelatinization, protein denaturation, browning, pore and crust formation, and shrinkage/swelling [7–9].

Chorizo is a Colombian meat product that is traditionally consumed in various forms. A cured raw sausage of Spanish origin, its preparation has spread throughout the world; its formulation and preparation vary according to country and even region [10, 11]. According to Colombian Technical Standard NTC 1325, chorizo is a fresh, raw, and processed meat product obtained by grinding or mincing cooked and matured meat, together with fat and other permitted substances [12]. Vacuum frying is a technological alternative that improves the conditions of the frying process, while maintaining the sensory properties and product quality [13]. This technology consists of immersing food in hot oil in an airtight container, where the process pressure is below the atmospheric pressure, so time is shorter and temperature is lower than that in atmospheric frying [14]. Operating at low process temperatures confers advantages such as preservation of flavor and nutrients, as well as protection of oil quality and reduction in the generation of toxic compounds [15–17].

Some studies have reported the use of mathematical models to describe, predict, and optimize the kinetic behavior of foods during the frying process. Fick's law has been used to describe the process of moisture diffusion and as a basis for optimizing processing conditions for different fried foods [18, 19]. Authors such as [20–24] applied this diffusive model during frying of pea chips, potato strips, yucca slices, peas and banana slices, and sweet potato slices, respectively.

Although frying is a commonly used process, its quantitative analysis in the engineering field is limited to food matrices of meat origin, where vegetable products are mainly studied to evaluate transport phenomena [25]. The use of mathematical models to predict mass transfer kinetics has been widely applied in food matrices of plant origin since there is a greater possibility of mass exchange, which would represent a greater adjustment to the mathematical model.

Suaterna [26] showed that foods of animal origin have tissues with spaces or intracellular pores that prevent the entry of oil (heating medium) during frying, while the intracellular space of plant tissues is full of air, allowing more oil collection. It is worth mentioning that different studies have been carried out for the effect of frying on fresh meat and some derived foods, including studies carried out by Candela et al. [27], Makinson et al. [28], and Sheard et al. [29].

There are also studies by Yildiz and Dincer [30] and Sobowale et al. [31] on sausage frying. In addition, snacks, such as potato chips or tortillas, are the most studied fried products, but information on transport phenomena in fried meat products is scarce. For this reason, this study looked at mass transfer kinetics during atmospheric and vacuum frying of a chorizo-type meat product. So far, there are no publications in the literature on immersion frying chorizo, making this study important since it will provide more scientific information on the kinetics of water loss and oil absorption during vacuum and atmospheric frying. Therefore, the objective of this study was to study mass transfer during vacuum and atmospheric frying of chorizo.

## 2. Materials and Methods

### 2.1. Raw Materials

The beef, pork fat, and vegetables were purchased from a local supermarket in the city of Cartagena. The other inputs were purchased from a specialized company (Insumos y Equipos JR S.A.S., Cartagena de Indias, Colombia). Palm vegetable oil with antioxidants (TBHQ or BHT) was used (Famar S.A, Santa Marta, Colombia). The oil was fresh and was replaced for all frying conditions.

### 2.2. Elaboration of Chorizos

Beef that was previously cleaned and weighed was used to make the chorizos. Subsequently, the grinding process was carried out with a 10 mm thick disc grinder. The grinding temperature of the process was not higher than 7°C to avoid protein denaturation [32].

The ground meat was weighed with the pork fat already chopped and ground (15% of the weight of the meat) for the calculation. In this way, the chorizo was formulated according to the concentration allowed by Colombian Technical Standard NTC 1325: beef (100%): pork fat (15%), ice (10%), sodium chloride (1.6%), pepper (0.5%), liquid smoke (0.15%), Welsh onion (5%), garlic (1.5%), nitrite (1.5%), polyphosphate (0.4%), and ascorbic acid (0.045%).

The kneading and mixing of the nonmeat raw materials (seasonings and additives) were carried out manually to obtain a homogeneous dough. Once the dough was obtained, the sausage was produced in a hydraulic stuffer, using natural casing; the tying was done manually. After the stuffing process, the sausages were subjected to a blanching process until the core of the sausages reached a temperature of ±72°C. Then, the sausages were immediately dried and subjected to heat shock. Finally, they were allowed to rest and refrigerated (4°C) for 16 h. Once the chorizos were prepared, the experiments were carried out.

### 2.3. Vacuum Frying

The frying of the samples under vacuum conditions was carried out with a Gastrovac® (Cocina Internacional, Barcelona), 40 × 26 × 46 cm, with a maximum capacity of 10.5 L. The maximum vacuum pressure used in the equipment was 30 kPa; the oil temperatures were 90°C, 120°C, and 150°C; the frying times were between 90 and 360 s, established by means of preliminary tests. 10 pieces of chorizo were used for each frying, 100 mm long × 15 mm wide, and the weight was 100 g approx. The food : oil ratio was 1000 g : 4 L.

To start the vacuum frying process, the chorizos were placed in a basket that was attached to the handle of the lid, the fryer and the vacuum valve were closed, pressure was applied to the lid to stabilize the vacuum pressure, and, once the pressure was reached, the samples were immersed in hot oil. The temperature and time were previously programmed. Once the frying process was finished, the samples were removed from the oil, the vacuum valve was opened to balance the pressure, and the samples were removed.

### 2.4. Atmospheric Frying

The frying at atmospheric pressure was done in a Professional Digital Waring Pro Df250b Fryer, 1800 Watts, with a capacity of 5 L. The frying times were between 90 and 360 s, and the oil temperatures were established by preliminary tests at 160°C, 170°C, and 180°C. The chorizos were drained into a wire mesh basket for further analysis. 10 pieces of chorizo were used for each frying, 100 mm long × 15 mm wide, and the weight was 100 g approx. The food : oil ratio was 1000 g : 4 L.

The frying by immersion was carried out in a discontinuous way: the fryer was connected to the electrical source, the temperature and time were established in the control panel, and vegetable oil was placed in a stainless steel container. Once the temperature was reached, the chorizos were placed in lots of three units in a mesh basket and immersed in the oil, and the lid was placed. The fried chorizos were left to rest for 5 min outside the fryer basket and passed through absorbent paper where they were drained for 5 min. This process was repeated three times with each temperature time, similar to Marcano et al. [33].

### 2.5. Determination of Moisture and Oil Content

The determination of moisture loss was calculated according to AOAC 925.10 [34], and the Soxhlet method was applied to determine the fat content with extraction with light petroleum (benzine) according to AOAC 920.85 [34]. All measurements were taken in triplicate.

### 2.6. Mathematical Modeling

A first-order kinetic model was chosen to describe the phenomena of mass transfer with concentration differences within the frying process, taking into account the following assumptions: (1) the oil temperature is constant during frying, (2) the initial concentration of water in chorizos is uniform, and (3) the two flows were considered independent of each other.

The effective diffusion coefficients for each temperature of the process were obtained from the analytical solution to Fick's second law for the geometry of the cylinder and in an unstable state, using constant diffusion.

### 2.7. Kinetics of Moisture Loss

To calculate the diffusion coefficients of the fried sausages in different processes (atmospheric and vacuum), a second-order mathematical model describing the mass transfer with concentration differences was used:

(1) $$\begin{array}{c} \frac{\partial C}{\partial t}=D_{a}\frac{\partial^{2}C}{\partial^{2}x^{2}}0\leq x\leq r,\;\text{for }0>t. \end{array}$$

The moisture concentration can be normalized according to the following equation [21]:

(2) $$\begin{array}{c} \text{MR}=\frac{M_{t}+M_{e}}{M_{o}+M_{e}\;}=\frac{\text{average final moisture content}}{\text{uniform initial moisture content}}. \end{array}$$

*M*_*t*_ is the moisture content in time *t*, *M*_*e*_ is equilibrium moisture content, *M*_*o*_ is for initial moisture content, and Fick's second law was used, described for the geometry of a cylinder proposed by Melquíades et al. [35]:

(3) $$\begin{array}{c} \frac{M_{t}+M_{e}}{M_{o}+M_{e}}=\frac{4}{5,783}e^{-5,783D_{a}t/r^{2}}. \end{array}$$

Assuming that *M*_*e*_ at time *t* is equal to zero (0), it was discarded. To determine the effective moisture diffusion coefficient (*D*_*a*_), the linearization of Equation (3) was done by applying logarithm and adjusting it to a straight line *y* = *mt* + *b*:

(4) $$\begin{array}{c} \text{Ln}\left(\frac{M_{t}}{M_{o}}\right)=-\frac{5.783D_{a}t}{r^{2}}+\text{Ln}\left(\frac{4}{5,783}\right). \end{array}$$

On the other hand, the influence of the frying temperature on the effective diffusion coefficient had an Arrhenius-type behavior according to the following equation:

(5) $$\begin{array}{c} D_{a}=D_{o}e^{-E_{a}/RT}. \end{array}$$

By linearizing the equation and applying the natural logarithm of the graph *LnD*_*a*_ vs. *T* (k), *E*_*a*_ was estimated from the value of the slope.

To determine the mass transfer coefficient of chorizos, Lewis' first-order model was used:

(6) $$\begin{array}{c} \text{MR}=-\exp^{k_{c}t}. \end{array}$$

*K*_*c*_ is mass transfer coefficient and *t* is time.

### 2.8. Kinetics of Oil Uptake

For the fat uptake modeling, a first-order kinetic model was used, which is the first-order kinetic model recommended by Krokida et al. [36],

(7) $$\begin{array}{c} O^{*}=O_{\text{eq}}\left(1-\exp^{-Kt}\right). \end{array}$$

*O*∗ is the oil content in time *t*, *O*_eq_ is the equilibrium oil content in time *t* = ∞, and *K* represents the specific rate of oil uptake for the first-order model. In the model, at *t* = 0, the oil content is zero, and, for longer times, the oil content reaches the value of equilibrium. Equation (7) was linearized, and a natural logarithm was applied on both sides. From the graph, the speed of oil uptake was calculated. This model gave acceptable accuracy between calculated and experimental values for all cases [37].

The relationship for the variation of equilibrium oil content, *O*_eq_, with frying temperature *T* can be described using an Arrhenius-type relationship to obtain the activation energy [38]:

(8) $$\begin{array}{c} A=A_{0}\exp\left(\frac{-E_{a}}{\text{RT}}\right). \end{array}$$

*A* is a reaction rate of the model parameters, *A*_0_ is the preexponential factor, *E*_*a*_ is the activation energy, *R* is the universal gas constant, and *T* is the absolute temperature. The linearization of Equation (8) and graph Ln *O*_eq_ vs. 1/*T* gave the linear slope, which allowed us to calculate the activation energy. To minimize errors, the root equation was used:

(9) $$\begin{array}{c} \text{RMS}=\sqrt{\frac{1}{N}}\overset{N}{\underset{i=1}{\sum }}{\left(\frac{V_{\exp}-V_{\text{fitted}}}{V_{\exp}}\right)}^{2}. \end{array}$$

In the above equation, *N* is the number of data points, *V*_exp_ is the experiment value, and *V*_fitted_ is the calculated value. Models based on Fick's law were adjusted to the experimental data.

### 2.9. Statistical Analysis

The data processing was carried out with the statistical program STATGRAPHICS Centurion 16.1.15 (Corporation, U.S.A.) on Windows 8. The existence or lack of statistically significant differences of each evaluated parameter was determined through a completely randomized analysis of variance (ANOVA) and multiple comparisons, at a significance level of 5% (*p* ≤ 0.05).

## 3. Results and Discussion

### 3.1. Kinetics of Moisture Loss

**Figure 1:**
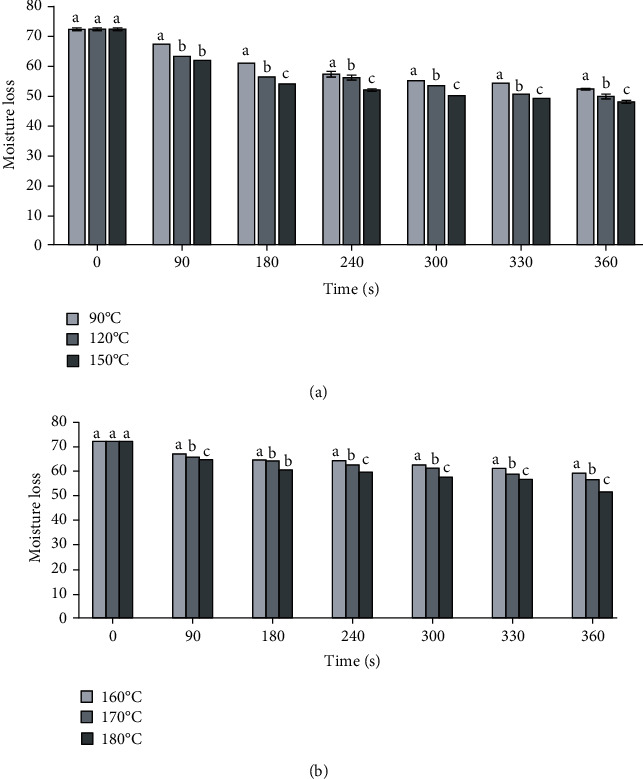
Moisture loss of chorizos vs. the frying time for each temperature: (a) vacuum frying and (b) atmospheric frying.

Figure 1 shows the kinetic movement of moisture loss from the chorizos during the vacuum and atmospheric frying processes. The curves between the different process temperatures presented a similar behavior, showing a direct relationship with time and temperature, i.e., as these variables increased, the moisture content of the chorizos during frying decreased (Figures 1(a) and 1(b)), with water evaporation being more noticeable at higher temperatures. The way in which a biological material loses water during frying is related to the physicochemical changes that occur in the cellular structure, which, in turn, are related to the processing temperature and time. This phenomenon was also observed by Sobowale et al. [31] during the frying of sausages, who stated that the moisture content of a meat product decreased with increasing temperatures and frying times. The application of higher frying temperatures saw a significant reduction in moisture content in both processes (vacuum and atmospheric).

In addition, the moisture content was reduced by 33.72% at a temperature of 150°C and 360 s of processing time during vacuum frying, as compared to atmospheric frying, which was 28.61%. When analyzing the lower temperatures and longer frying times in both processes, the frying of chorizos at atmospheric pressure presented an average of 11% lower final moisture content (*p* < 0.05). Likewise, the moisture content of the chorizos at lower vacuum frying temperatures, independent of the processing time, was similar to the moisture content of the chorizos at atmospheric temperature, which means that it is possible to obtain chorizos with the same moisture content by applying lower process temperatures under vacuum conditions.

At intermediate vacuum temperatures (90°C) and a time of 240 s, the moisture content of the chorizos was similar in atmospheric conditions (170°C, 360 s) (*p* < 0.05); once again, the vacuum frying had similar moisture contents in shorter processing times. These results were obtained by Faloye et al. [39] for chicken nuggets, demonstrating that the moisture content decreased significantly with increasing temperatures and frying times, regardless of the thickness of the sample used. Previous studies on chorizo frying were carried out by Acevedo et al. [40], demonstrating that the factor that most influenced the dehydration of chorizos after vacuum frying was temperature, which in combination with processing time directly influences moisture loss.

Table 1 shows that the behavior of the diffusion coefficient in sausages fried at atmospheric pressure and under vacuum was higher when higher process temperatures were applied. Also, during atmospheric frying, this parameter was lower under atmospheric conditions than that seen with the vacuum application. That is, the effect of vacuum (30 kPa) produced expansion in the pores of the chorizo, which mobilized free water molecules faster because of the decrease in the boiling point of water.

Yildiz and Dincer [30] obtained lower diffusion coefficients (4.101 × 10^−9^ m^2^/s) during the frying of cylindrical sausages at 180°C. These authors stated that temperature has an enormous influence on diffusion because of the driving force for mass transfer provided by the conversion of water into steam, which is forced out of the pores of the matrix.

Similar results were reported by Nasiri et al. [41], who stated that the effective moisture diffusivity of shrimp nuggets ranged from 2.05 × 10^−8^ to 5.71 × 10^−8^ m^2^/s with *R*^2^ between 0.91 and 0.98. The frying temperature had a positive effect on the effective diffusivity of moisture, increasing with increasing oil temperatures. Sosa-Morales et al. [42] obtained lower diffusion coefficients during pork frying (plate shape), within the range of 1.5 × 10^−9^ to 30.2 × 10^−9^ m^2^/s, estimated between 90 and 110°C. The observed differences in moisture diffusivity could have been due to the effect of the different formulation of the chorizo and different food ingredients.

Osorio et al. [20] agreed with the effect of pressure on moisture diffusivity in fried peas at 140, 160, and 180°C, obtaining values between 1.62 ± 0.12 and 2.61 ± 0.23 × 10^−8^ m^2^/s for 78 kPa, 2.01 ± 0.24 and 2.86 ± 0.16 × 10^−8^ m^2^/s for 43 kPa, and 2.42 ± 0.18 and 3.44 ± 0.31 × 10^−8^ for 9 kPa m^2^/s. These authors indicated that the higher the vacuum, the higher the drying speed, and the shorter the processing times. An increase in pressure results in low diffusivity values; that is, at moderately low pressures, the diffusion coefficient of gases is inversely proportional to pressure or density. This fact is attributed to variation in the density of gases.

On the other hand, as the pressure decreases, the boiling point of the water decreases, and, as a result, the water contained in the product will start to vaporize faster from the inside of the food. This explains the values obtained in the convective coefficient or rate of water loss, where its dependence on temperature was observed. Higher values were seen with vacuum frying because of the effect of pressure, which indicates a higher speed of vaporization [22].

For the moisture loss kinetic constants, it was observed that they increased with increasing temperatures in both types of frying; however, the vacuum frying was higher on average by 36%. Similar data were reported by Barrios et al. [43] with calculated *K*_*c*_ coefficients during pea frying at temperatures of 160, 180, and 200°C, reporting maximum values of 1.25 × 10^−2^, 1.44 × 10^−2^, and 1.94 × 10^−2^ m/s.

**Figure 2:**
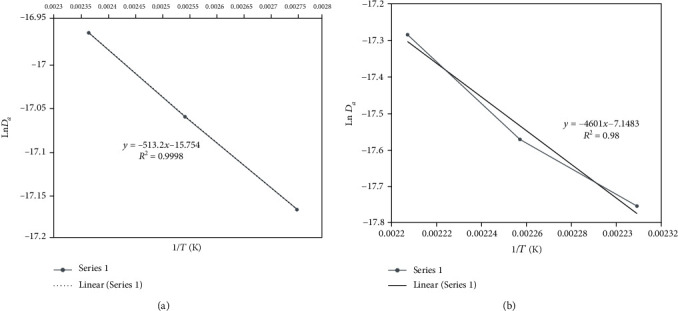
Logarithm of effective moisture diffusivity vs. absolute frying temperature: (a) vacuum frying and (b) atmospheric frying.

The activation energy (*E*_*a*_) of the immersion frying operation was determined from the diffusion coefficient that fit the Arrhenius equation because of dependence on temperature [43].  Figure 2 shows the relationship with Ln *D*_*a*_ (m^2^/s) as a function of 1/*T* (K^−1^), where the value of the activation energy with the slope of the line was calculated. Sobowale et al. [31] reported activation energies of 71.04 to 77.76 kJ/mol and 65.82 to 67.2 kJ/mol for temperatures of 150, 170, and 190°C, respectively, for frying goat meat sausages. The activation energy increased significantly with increasing temperatures. The lower activation energies indicated that sausage frying requires less energy and that the process is less sensitive and dependent on temperature changes [44].

The vacuum (pressure of 30 kPa) and lower temperatures (of 150°C) resulted in an energy requirement to start the diffusion process that was much lower than that with normal pressures (1 atm). The activation energy (*E*_*a*_) was 4.3 kJ/mol for the vacuum pressure and 38.25 kJ/mol for the atmospheric pressure, with determination coefficients of 0.99 and 0.98, respectively (Table 1). Ortega and Montes [22] reported activation energies (*E*_*a*_) of 70.9424 kJ/kmol (*R*^2^ = 0.9905) for a pressure of 78 kPa and 23.2 kJ/kmol (*R*^2^ = 0.8673) for a pressure of 9 kPa, with frying temperatures of 140, 160, and 180°C for atmospheric pressure and 90, 110, and 130°C for vacuum pressure, with a time of 1000 s for both pressures. Barrios et al. [43] indicated that the activation energy of this phenomenon comes from the thermal energy of the fluid (oil), observing that, as the system is emptied, less *E*_*a*_ is required to start the process.

### 3.2. Kinetics of Oil Uptake

**Figure 3:**
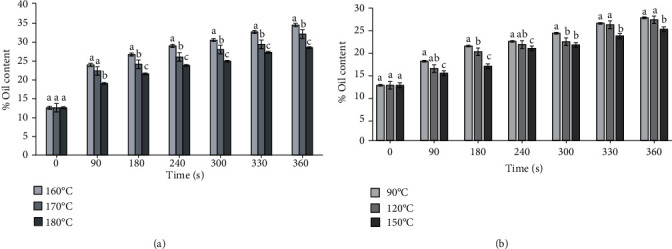
Kinetics of oil uptake: (a) vacuum frying and (b) atmospheric frying.

The oil content at the end of the process (360 s) was 27.79% (90°C), 27.31% (120°C), and 24.82% (150°C) for the vacuum-fried chorizos, and higher values were obtained in the atmospheric frying, obtaining values of 34.45% (160°C), 31.36% (170°C), and 28.47% (180°C) (*p* < 0.05). Also, during the atmospheric frying at 180°C and maximum frying time, the final oil content in the chorizos was similar to the content with a frying temperature of 160°C and time of 240 s. In addition, at the maximum processing temperature and time, a lower oil percentage, 21.00% and 11.20%, was obtained than that with temperatures of 160°C and 170°C, respectively (Figure 3(a)) (*p* < 0.05).

The maximum difference between the experiment and regression values was 1.21% for the atmospheric frying and 1.23 for the vacuum frying, demonstrating excellent agreement between the data (Figures 3(a) and 3(b)). These results were similar to those of Sobowale et al. [31] for goat meat sausage frying, who reported higher oil contents with increasing frying temperatures and times, going from 19.38% to 36.0 2% at 170°C, 3 min and 9 min, respectively.

On the other hand, the vacuum frying at temperatures of 90°C and 120°C presented the same behavior for the fat absorption kinetics. On the other hand, at higher process temperatures (150°C), the final oil percentage was lower on average by 11% than that with the other two temperatures (90°C and 120°C) (Figure 3(b)). Yildiz and Dincer [30] also reported this same trend in frying cylindrical sausages, stating that the oil content increased with increasing the frying time. Faloye et al. [39] also demonstrated that oil content increased when the frying temperature increased from 155 to 175°C for 5 min in chicken nuggets. Thus, at higher temperatures, there tends to be faster development of the solid crust, which increases oil absorption, especially during cooling.

In general, it was found that fat absorption increased, while moisture content was significantly reduced in both frying types (atmospheric and vacuum). As the temperature decreased for a processing time, the oil content increased in the chorizos. For the vacuum frying, when the oil uptake decreased from 90°C to 150°C with a time of 240 s, the same behavior was shown for frying at atmospheric pressure.

Teruel et al. [45] reached the same conclusion when they studied the vacuum effect on chicken nugget frying. This can be explained by the phenomenon of pressurization that occurs after frying and before the cooling stage. Once the food is removed, the internal pressure of the pores begins to increase until equilibrium with the external environment is reached, at which time the oil and air (gas) on the surface of the food begin to penetrate the empty spaces [46].

Because of the difference in pressure at that time, the diffusivity of air is much higher than that of water, and, therefore, air begins to occupy those spaces, preventing oil from entering the food; in the cooling stage, like in atmospheric frying, oil located on the surface of food continues to penetrate the pores in vacuum frying, with the difference that, with this technology, oil adheres to a lesser degree on the surface of the product, so there is less oil available to penetrate during this stage [47].

There are some factors that influence oil uptake in foods, such as the quality and composition of oil, surfactants produced by oxidation, frying temperature, shape of the food, moisture content, and porosity and process conditions [22].

Bermúdez et al. [23] used the same temperatures used in this study under atmospheric conditions and reported similar results when frying banana slices. Barrios et al. [43] reported that, at a temperature of 160°C, the highest uptake value of 0.343 g oil/g dry solid was seen, which decreased by 9.621% at 180°C and 18.367% at 200°C.

A lower fat content was observed during the vacuum frying. A similar behavior was found by Urbano et al. [48] who carried out vacuum frying at 100, 120, 130, and 140°C and compared it with atmospheric pressure (165°C), indicating that using reduced pressures means the oil uptake decreases.

### 3.3. Parameters of Oil Uptake Kinetics

Table 2 shows the parameters describing the oil uptake kinetics for the vacuum and atmospheric frying. The temperature had an inverse and significant effect on the rate of oil uptake, i.e., a decrease in the rate of oil uptake was seen with increasing temperatures. The *K* values ranged from 4.7 × 10^−3^ at 90°C (*R*^2^ = 0.99) to 4.5 × 10^−3^ s^−1^ (*R*^2^ = 0.88) at 150°C (under vacuum conditions) and from 6.4 × 10^−3^ at 160°C (*R*^2^ = 0.93) to 6.4 × 10^−3^ s^−1^ (*R*^2^ = 0.86) at 180°C (under atmospheric conditions).

A different behavior was seen by Nasiri et al. [41] during shrimp nugget frying, obtaining an increase in a range between 3.5 × 10^−3^ and 7.8 × 10^−3^ s^−1^. It should be noted that the rate of oil uptake depends on the main process variables, such as oil temperature, type of product, pretreatment applied, frying conditions, product width, and type of oil [29, 32, 49]. In addition, the decrease in the speed constant was due to the rapid formation of the crust at high temperatures, a fact that would explain the results found for chorizos. The values of the root mean square (%RSM) and the coefficient of determination *R*^2^ indicated a good fit of the kinetic model with the experiment data. For the calculation of the activation energy, the *O*_eq_ values were used for the maximum oil content uptake by the chorizos under different frying conditions and the maximum time used in the experiment. The oil content at equilibrium (${\underset{¯}{O}}_{\text{eq}}$) decreased when the oil temperature increased. This result agrees with the results of [22, 41], who studied mass transfer parameters for shrimp and nugget frying of yucca, respectively. The negative activation energy values for the oil uptake showed a decreasing trend with increasing temperatures. Similar results were reported by Ortega and Montes [22] for nugget frying of yucca slices, obtaining values of −10.25 ± 2.52, −2.77 ± 0.66, and −17.22 ± 3.75 kJ/mol for control and blanched and osmodehydrated samples.

## 4. Conclusion

Both frying types had a moisture content in the sausages that decreased gradually as the temperature and processing time increased. In addition, the moisture content during vacuum frying was reduced by 33.72% at the maximum temperature and processing time, as compared to the atmospheric frying, which was 28.61%. The vacuum frying had similar moisture contents in the shorter processing times. Also, the behavior of the diffusion coefficient in the sausages fried at atmospheric pressure and under vacuum was higher when higher process temperatures were applied. In addition, this parameter was lower under atmospheric conditions. Similarly, at the maximum process temperature and time (180°C and 360 s), a lower percentage of oil was obtained than that with the temperatures of 160°C and 170°C, 21.00% and 11.20%, respectively (Figure 3(a)).

## Figures and Tables

**Table 1:** Moisture transfer parameters of vacuum and atmospheric pressure fried chorizos, diffusivity, and convective moisture coefficient.

Type of frying	*T* (°C)	*D*_*a*_ (m^2^/s)	*K*_*c*_ (m/s)	*R* ^2^	*E*_*a*_ (kJ/mol·K)
Vacuum frying	90	3.50 × 10^−8^	9 × 10^−4^	0.98	4.3
	120	3.89 × 10^−8^	10 × 10^−4^	0.97	
	150	4.28 × 10^−8^	11 × 10^−4^	0.95	

Atmospheric frying	160	1.95 × 10^−8^	5 × 10^−4^	0.95	38.25
	170	2.33 × 10^−8^	6 × 10^−4^	0.95	
	180	3.11 × 10^−8^	8 × 10^−4^	0.94	

**Table 2:** Parameters describing oil uptake kinetics of chorizos and activation energy with Arrhenius type fitting.

Frying type	*T* (°C)	Oil absorption speed				Calculation of the activation energy			
		*K*(s^−1^ × 10^−3^)	*R* ^2^	%RSM	1/*T* (K^−1^∗10^−3^)	*O*_eq_	Slope (m)	*R* (kJ mol/K)	*E*_*a*_ (kJ/mol)
Vacuum frying (30 kPa)	90	4.7	0.99	0.10	2.75	27.79	283.25	8.31	-2.35
	120	3.8	0.98	0.109	2.54	27.31			
	150	4.5	0.88	0.069	2.36	24.83			

Atmospheric frying	160	6.4	0.93	0.095	2.31	34.45	1871.8	8.31	-15.54
	170	6.0	0.93	0.132	2.26	31.36			
	180	6.4	0.86	0.091	2.21	28.47			

## Data Availability

The data used as references in this study have been indicated in the reference repository (name of authors, year of publication, title of the document, journal, and number of pages).
